# What Has Been Studied About Attitudes and Social Stigma Towards HIV/AIDS? A Global Bibliometric Study with Correlations on Global Health HIV-Related Indicators

**DOI:** 10.3390/healthcare13080891

**Published:** 2025-04-13

**Authors:** Yelson Alejandro Picón-Jaimes, Ivan David Lozada-Martinez, Mar Rosàs Tosas, Juan Tiraboschi, Ornella Fiorillo-Moreno, Valmore Bermúdez

**Affiliations:** 1 Facultat de Ciències de la Salut Blanquerna, Universitat Ramon Llull, 08025 Barcelona, Spain; mariadelmarrt1@blanquerna.url.edu; 2 Biomedical Scientometrics and Evidence-Based Research Unit, Department of Health Sciences, Universidad de la Costa, Barranquilla 080002, Colombia; ilozada@cuc.edu.co; 3 Unitat de VIH, Servei de Malalties Infeccioses, Hospital Universitari de Bellvitge-IDIBELL, Universitat de Barcelona, 08028 L’Hospitalet de Llobregat, Spain; jmtiraboschi@bellvitgehospital.cat; 4 Clínica Iberoamérica, Barranquilla 080001, Colombia; ornella.fiorillo11@gmail.com; 5 Clínica El Carmen, Barranquilla 080001, Colombia; 6 Centro de Investigaciones en Ciencias de la Vida, Facultad de Ciencias de la Salud, Universidad Simón Bolívar, Barranquilla 080001, Colombia

**Keywords:** HIV, acquired immunodeficiency syndrome, social stigma, microaggression, health knowledge, attitudes, global health

## Abstract

**Introduction:** This study aimed to assess, through health metrics and bibliometric analysis, the global research on attitudes and social stigma of people living with HIV/AIDS and to identify research findings, gaps, and future directions. **Methods:** A cross-sectional bibliometric study was conducted through a structured search in different databases. Fifteen thousand four hundred and ninety-six documents were found between 1981 and 2024. **Results:** 83.5% were original articles, and international co-authorship was 30.66%. Since 2000, there has been an increase in research on HIV/AIDS attitudes and social stigma. The United States is the most prolific country worldwide (n = 7837 publications; 50.5%), with the highest number of prolific institutions (n = 4/5), as well as the greatest influence and relevance in research (h-index 170). The most studied topics worldwide are social support and social psychology concerning homosexuality, middle age, and youth in people living with HIV/AIDS. There was no significant correlation between the volume of publications, countries’ income levels, and the most prolific geographic regions with adult HIV prevalence, overall HIV incidence and prevalence, or antiretroviral therapy coverage in people living with HIV (*p* > 0.05 for all cases). **Conclusions:** Over the past two decades, research has shifted from human rights, legal rights, and ethics to attitudes toward healthcare, with the recent interest in pre-exposure prophylaxis, gender minorities, and intersectional stigma. The absence of strong correlations between publications volume and global health HIV-related indicators underscores the necessity of translating evidence into actionable strategies to reduce stigma and improve health outcomes.

## 1. Introduction

The stigmatisation of people living with the human immunodeficiency virus (PLWH) and acquired immunodeficiency syndrome (AIDS) remains a widespread and deep-rooted problem that leads to discrimination and hinders access to adequate healthcare systems [1,2,3]. Over the years, numerous campaigns have been launched by researchers, healthcare providers, and advocacy groups to combat the stigma and understand better the complexities of living with human immunodeficiency virus (HIV) [1,2,3].

Despite decades of efforts, HIV-related stigma persists globally, fuelled by cultural, social, and economic factors. Meta-analyses highlight persistent gaps in interventions targeting structural stigma, gender minorities, and intersectional discrimination [4,5]. These studies emphasise the importance of integrating socio-behavioural research, public health strategies, and legal reforms to dismantle stigma effectively [4,5]. However, health metrics demonstrate that disparities remain, particularly in low- and middle-income countries, where healthcare inequities and socioeconomic barriers compound stigma.

The stigmatisation of PLWH manifests itself in various ways, such as social rejection, familial discrimination, violence, and lack of access to health services [6,7,8]. These negative and stigmatising attitudes can have a significant impact on people’s quality of life and emotional and mental well-being [6,7,8]. It is important to emphasise that stigma results from a lack of knowledge and misinformation [6,7,8]. It is often associated with damaging myths and stereotypes, such as the belief that HIV is a disease reserved for certain social groups or minorities [9,10,11]. Tackling the stigmatisation of PLWH requires a multi-dimensional approach that includes education, the promotion of human rights, and access to healthcare and support services. It is essential to promote empathy to combat entrenched stereotypes and prejudices.

The broader research landscape reflects the growing interest in understanding HIV-related stigma through sociocultural, psychological, and epidemiological lenses. Studies have evolved to address issues of internalised stigma, microaggressions, and the role of healthcare providers in perpetuating discriminatory practices [4,5]. Furthermore, recent research highlights the need for intersectional approaches that examine stigma across gender, sexual orientation, and socioeconomic contexts [4,5].

In addition, research on stigma needs to be promoted and sufficiently disseminated so that professionals, healthcare decision-makers, and the public recognise the issue [9,10,11]. Looking at the past and knowing the progress made in defining this problem is necessary to address stigma adequately. This view creates a solid foundation for initiating new lines of research to help implement procedures to reduce stigma [12]. Through health metrics and bibliometric analysis, this study aimed to assess the global research on attitudes and social stigma towards PLWH and identify research findings, gaps, and future directions.

## 2. Methods

### 2.1. Study Design

A cross-sectional bibliometric study was conducted. This type of study consists of a quantitative analysis of the scientific literature using mathematical and statistical methods to evaluate and analyse the production and impact of publications. This type of study allows for identifying trends, collaboration patterns, and the evolution of research topics over time.

In addition, it can include analysing co-authorship and citation networks, observing temporal trends that allow us to see how a topic evolves, and measuring the impact of research in the scientific community.

Unlike other methodologies, such as systematic reviews and narrative reviews, bibliometric studies focus on quantitative analysis. In contrast, systematic reviews follow a rigorous protocol and seek to collect and synthesise all relevant evidence on a specific research question.

The usefulness of bibliometric studies in research on HIV stigma is significant. These studies can identify trends in research, influential authors, and areas that require more attention. For example, they can reveal how perceptions of stigma have changed over time and what approaches have been most effective in research and intervention. They can also help identify gaps in the literature and emerging areas that need further exploration.

### 2.2. Source Database

Scopus, Web of Science, Medline, Scielo, and PsycINFO databases were used to conduct an exhaustive documentary search. These are the largest databases of peer-reviewed literature, indexing the most relevant journals and publications in health literature [13,14].

### 2.3. Search Strategy

A search was conducted to identify all published documents worldwide regarding this topic, and it reviewed MeSH terms and synonyms to search.

The search formula designed and used was: TITLE-ABS-KEY(HIV) OR TITLE-ABS-KEY(“Human Immunodeficiency Virus”) OR TITLE-ABS-KEY(“Human Immunodeficiency Viruses”) OR TITLE-ABS-KEY(“AIDS Virus”) OR TITLE-ABS-KEY(“AIDS Viruses”) OR TITLE-ABS-KEY(“Acquired Immune Deficiency Syndrome Virus”) OR TITLE-ABS-KEY(“Acquired Immunodeficiency Syndrome Virus”) OR TITLE-ABS-KEY(“Acquired Immune Deficiency Syndrome”) OR TITLE-ABS-KEY(“Acquired Immuno-Deficiency Syndrome”) OR TITLE-ABS-KEY(“Acquired Immunodeficiency Syndrome”) OR TITLE-ABS-KEY(“Acquired Immuno-Deficiency Syndromes”) OR TITLE-ABS-KEY(“Acquired Immunodeficiency Syndromes”) OR TITLE-ABS-KEY(AIDS) OR TITLE-ABS-KEY(“HIV Seropositivity”) OR TITLE-ABS-KEY(“AIDS Serodiagnosis”) OR TITLE-ABS-KEY(“HIV Seroprevalence”) OR TITLE-ABS-KEY(“AIDS Dementia Complex”) OR TITLE-ABS-KEY(“Acquired Immunodeficiency Syndrome”) OR TITLE-ABS-KEY(“Acquired Immunodeficiency Syndromes”) AND TITLE-ABS-KEY(“Social Stigma”) OR TITLE-ABS-KEY(“Social Stigmas”) OR TITLE-ABS-KEY(Stigma) OR TITLE-ABS-KEY(Prejudice) OR TITLE-ABS-KEY(Homophobia) OR TITLE-ABS-KEY(Microaggression) OR TITLE-ABS-KEY(Aggression) OR TITLE-ABS-KEY(Dehumanization) OR TITLE-ABS-KEY(Harassment) OR TITLE-ABS-KEY(“Non-Sexual Harassment”) OR TITLE-ABS-KEY(Bullying) OR TITLE-ABS-KEY(“Psychology Rejection”) OR TITLE-ABS-KEY(“Social Discrimination”) OR TITLE-ABS-KEY(“Sexual Harassment”) OR TITLE-ABS-KEY(“Social Marginalization”).

Documents meeting the following criteria were included in the analysis: (1) Scientific documents subjected to the standard peer-review process and published in scientific journals with regular serial publications; and (2) Documents with a general and explicit objective related to analysing, discussing, investigating, summarising, or examining attitudes and social stigma towards PLWH. Documents meeting at least one of the following criteria were excluded: (1) conference proceedings, book chapters, books, errata, and retracted documents; and (2) documents lacking basic bibliographic information (e.g., author details, journal name, correspondence information).

Additionally, documents published in English or Spanish were included. Also, documents not initially published in English or Spanish but including an abstract in one of these languages were included, provided they met all inclusion criteria and none of the exclusion criteria. All countries’ output was considered from 1981 to 2024, marking the reporting of the first HIV cases [15].

### 2.4. Standardisation and Data Collection

This search was conducted until 30 September 2024, using the filters “human” and “journals”. Two authors (YAP and ILM) performed a manual review, eliminating duplicates and irrelevant articles based on title, abstract, and keywords in Microsoft Excel^®^.

Subsequently, two authors (YAP & ILM) reviewed the documents that were ultimately selected for data refinement and standardisation. The “articles” typology included original studies. The “Review” typology encompassed narrative reviews, systematic reviews, and meta-analyses. The “Editorial” typology included any article published under this category. The “Letter” typology encompassed other types, such as comments, notes, and correspondence.

Despite specifying that one of the inclusion criteria was that only peer-reviewed documents would be included, it is important to note that most journals indexed in the consulted databases implement a peer-review process, as this is one of the indexing requirements. However, documents classified under “Editorial” and “Letter” do not always meet this peer-review standard and may only undergo editorial review.

### 2.5. Evaluated Indicators and Metrics

To evaluate the influence of scientific production, we calculated the h-index, m-index, and g-index, along with the frequency of citations. The h-index quantifies the impact of scientific output based on the number of citations received by published articles [16,17]. The m-index, or Hirsch’s m-quotient, measures the linear correlation of an investigator’s impact over time. Finally, the g-index provides an additional quantitative measure derived from the distribution of an author’s accumulated citations (g-value), organised to correspond to the g^2^ ranking in descending order [16,17].

To determine the relevance and pertinence of global research, the World Health Organization (WHO) Global Health Observatory indicators related to the HIV epidemic and response were explored for comparison with the publication volume of the most prolific countries [18]. The available and most updated values for each country were used. The definitions and specifications of each indicator are open access and available to the public [18].

### 2.6. Data Analysis and Visualisation

We employed bibliometric and network metrics to assess the selected articles. Quantitative bibliometric indicators were computed using the bibliometrix package in R (Version 4.3.1) [19]. The variables relating to the characteristics of the studies and authors, including frequency and percentage calculations, were analysed using Microsoft Excel^®^.

Our research also delved into the characterisation of scientific growth and output. We applied Lotka’s law to illustrate the distribution of publications among authors and production by affiliation and country. Furthermore, we outlined studies with the highest impact, a crucial aspect of our research, and visualised the most studied topics worldwide. Collaboration networks between countries and authors through affiliations were established, providing valuable insights into the global scientific community.

Finally, multiple regression analyses and Pearson’s or Spearman’s correlation coefficient tests were used to evaluate potential associations and correlations between the most prolific countries, publications, and global health HIV-related indicators. For this analysis, the countries were further clustered according to their income level (low-income, lower-middle income, upper-middle income, and high-income economies) and the geographic region to which they belonged (African Region, Region of the Americas, Southeast Asia Region, European Region, Eastern Mediterranean Region, and Western Pacific Region). A *p*-value < 0.05 was considered statistically significant. All analyses were performed using the R statistical package.

### 2.7. Ethical Statement

This study was approved by the Clinical Research Ethics Committee (CEIC) of the Bellvitge Hospital (Catalonia) on 25 January 2024 (Minute 03/24), Ref. PR352/23.

## 3. Results

### 3.1. General Characteristics and Annual Growth

We initially found 16705 documents. After the authors manually reviewed and verified the inclusion criteria, 15496 documents were left from 1981 to 2024. 83.5% (n = 12,948) were original articles, while 9.5% (n = 1466) were review papers.

The research community’s dedication to HIV/AIDS research is evident concerning the number of authorships, which stands at 40403. This collaborative effort is further highlighted by the international co-authorship rate of 30.66% and an average of 4.73 authors per article. The annual scientific growth rate was approximately 9%, and the average number of citations per paper was 20 (see Table 1).

**Figure 1:**
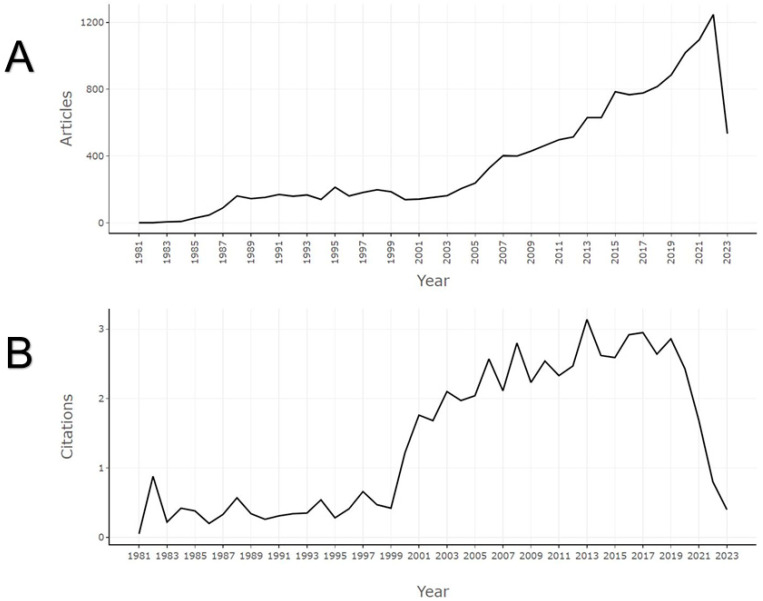
Annual scientific growth of global research on attitudes and social stigma towards HIV/AIDS. (**A**) Annual production volume. (**B**) Average yearly received citations.

Since the 2000s, research on HIV/AIDS attitudes and social stigma has experienced a significant emergence, with 2022 marking the pinnacle of productivity (>1200 articles) (see Figure 1A). A similar trend can be observed regarding citations, with 2013 being the year with the highest number of citations (see Figure 1B).

Applying Lotka’s law to analyse author productivity, we found that 73% of authors published only a single article, followed by 13.8% who published two.

### 3.2. Journals

**Figure 2:**
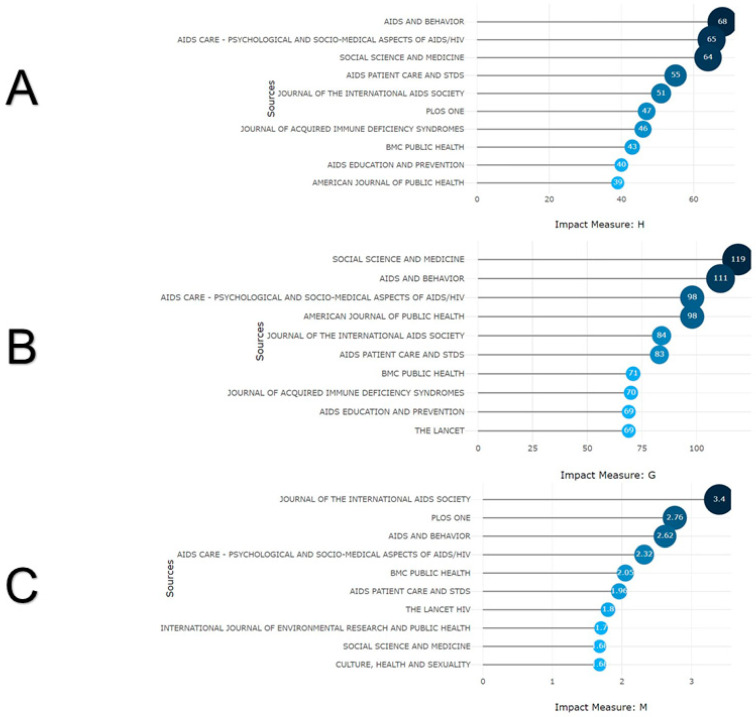
Journals impact of their articles on attitudes and social stigma towards HIV/AIDS. (**A**) h-index derived from the articles. (**B**) g-index derived from the articles. (**C**) m-index derived from the articles.

It was evident that “AIDS Care-Psychological and Socio-Medical Aspects” (n = 799; 5.1%), “AIDS and Behaviour” (n = 679; 4.3%), and “AIDS Policy & Law” (n = 439; 2.8%) were the journals with the highest number of published documents. However, when calculating the impact of journals derived from these publications, it was found that “AIDS and Behaviour”, “AIDS Care -Psychological and Socio-Medical Aspects”, and “Social Science and Medicine” have the highest h-index (h-index 68, 65, and 64, respectively) (Figure 2A). However, according to the g-index, “Social Science and Medicine”, “AIDS and Behaviour”, and “AIDS Care-Psychological and Socio-Medical Aspects” lead this metric (g-index 119, 111, and 98, respectively) (Figure 2B). As for the m-index and total citations, the “Journal of the International AIDS Society” (m-index = 3.7), “PLoS ONE” (m-index = 2.7), “AIDS and Behaviour” (n = 21,046), and “AIDS Care-Psychological and Socio-Medical Aspects” (n = 20,397) have had the most significant influence (Figure 2C). Annually, the previously mentioned journals maintain their prestige as the most sought-after publications on this topic, with a minimum annual publication of approximately 40 papers.

### 3.3. Affiliations

The top five consisted of four affiliations from the United States and one from Canada. The University of California (n = 1001), Johns Hopkins University (n = 741), and the University of Washington (n = 606) were the institutions with the most papers; however, to consider the h-index, the University of California, Johns Hopkins University, and Columbia University are the ones that have had the most significant impact (h-index of 95, 70, and 66, respectively). Since 1981, all previous institutions experienced a slow rise in publications until 2000, with a notable increase in research output until 2010 and a drastic ascent until 2024 (four times major) (Table 2). When assessing the collaboration network among institutions, a strong collaboration was evident among American institutions and a weaker collaboration among European institutions.

### 3.4. Countries

**Figure 3:**
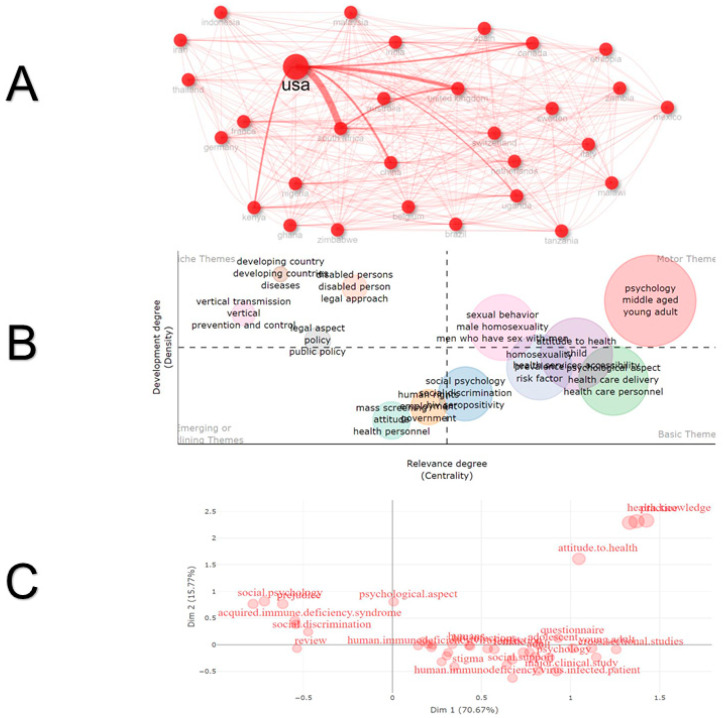
Collaboration networks, thematic map and multiple correspondence analysis on attitudes and social stigma towards HIV/AIDS. (**A**) Countries collaboration. (**B**) Thematic map and hot topics degree and status. (**C**) Factorial analysis of keywords word map.

In this case, the United States, the United Kingdom, and South Africa were the countries that have conducted the most research on attitudes and social stigma towards HIV/AIDS (n = 7837, 1716, and 1496, respectively), and their h-indices were 170, 94, and 86, respectively. Although all countries experienced slow growth in publications until 2000 (except the USA, which had published 535 papers by that time), there was a significant increase in publication volume from 2010 onwards compared to the previous 30 years (Table 2). When visualising the network and strength of collaboration between countries, a robust network was found between the most productive countries, led by the United States (Figure 3A).

### 3.5. Hot Topics and Research Outputs

The most frequently used keywords were “social psychology”, “social discrimination”, “sexual behaviour”, and “homosexuality”; among titles and abstracts, notable words included “middle age”, “young adult”, “social support”, “qualitative research”, and “homosexuality”. Analysing the evolution of topics from 1981 to 1990, legal approaches, legal rights, and homosexuality were principal topics; from 1991 to 2000, psychosocial study, minorities, politics, ethics, health education, and AIDS were of interest; from 2001 to 2013, topics primarily included vertical transmission and healthcare delivery, HIV in children, stereotyping, counselling, health personnel attitudes, and socioeconomic factors; finally, from 2013 to 2024, the topics of interest have evolved to research on intersectional stigma, gender minorities, pre-exposure prophylaxis, mental health and social support, antiretroviral therapy, and monkeypox.

Through co-occurrence networks and multiple correspondence analyses, we found that social stigma related to quality of life, healthcare, and antiretroviral therapy was the topic most frequently studied. In terms of topic degrees, social psychology, general discrimination, healthcare delivery, and HIV/AIDS-related risk factors have been studied as foundational themes. These themes are often combined with themes about sexual behaviours and young adults, with an emerging focus on research in developing countries (Figure 3B). However, as research gaps, it can be observed that the study of knowledge and health attitudes, including stigma experienced by health personnel or stigma for caregivers with HIV/AIDS, is isolated from the rest of the research outputs. Predominantly, global research on attitudes and social stigma towards HIV/AIDS has focused on questionnaires (contribute: 2.72), qualitative approaches (contribute: 0.46), and cross-sectional studies (contribute: 9.55) (Figure 3C). Dimension 1 exhibited a range of values between −0.31 and 2.53, while Dimension 2 ranged from −0.84 to 1.86. The mean value for Dimension 1 was 0.61, and for Dimension 2, it was 0.22. This indicates that the keywords are slightly dispersed along Dimension 1 but more concentrated along Dimension 2. Calculating the Euclidean distance between words revealed considerable variability among concepts, with a range from 0.77 to 2.48. A negative correlation (r^2^ = −0.30) was observed between the dimensions, which was not statistically significant (*p* = 0.18), suggesting independent patterns of variability.

### 3.6. Most Relevant Studies

Analysing the impact and characteristics of the ten most relevant studies in this field [20,21,22,23,24,25,26,27,28,29], the United States authored all of them, with the participation of Uganda, South Africa, and the United Kingdom, in three articles. The timeframe for these studies ranges from 2001 to 2013, with the most cited document titled “HIV and AIDS-related stigma and discrimination: a conceptual framework and implications for action” (1705 citations), followed by “Measuring stigma in people with HIV: Psychometric assessment of the HIV stigma scale” (1080 citations). The typology and approach of these documents are diverse, including original studies and reviews (Table 2). Ninety percent of these documents were published in Q1 journals.

### 3.7. Relevance of Research on Social Stigma and Attitudes Towards HIV According to Global Health HIV-Related Indicators

Exploring HIV-related indicators within the context of global health reveals countries and regions worldwide with the highest HIV burden and examines their relationship with the historical volume of publications. This analysis specifically focuses on the ten most prolific countries in research on social stigma and attitudes toward HIV (in descending order: USA, UK, South Africa, Canada, Australia, China, India, Uganda, Kenya, and Brazil), highlighting notable correlations.

**Figure 4:**
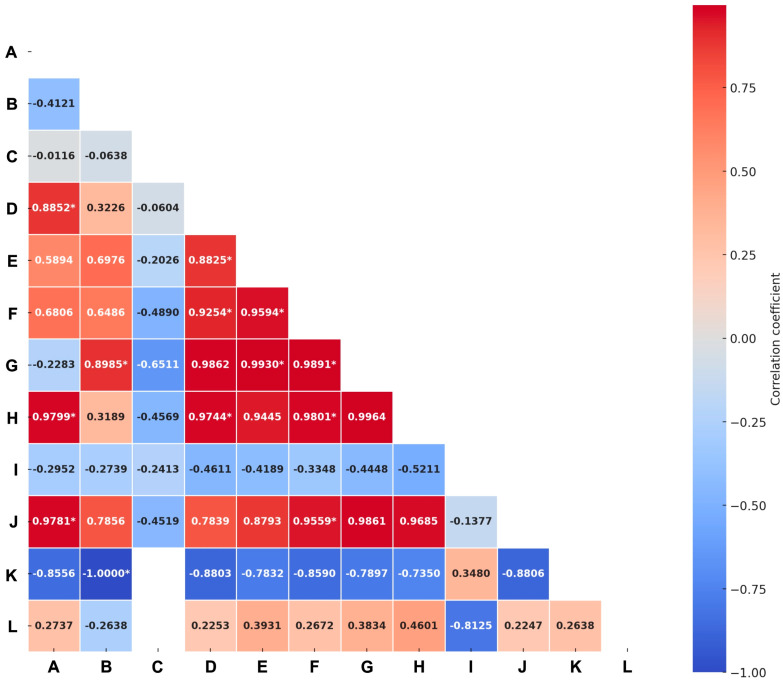
Correlations between the volume of publications on attitudes and social stigma towards HIV/AIDS and global health HIV-related indicators. Empty data represents insufficient information for the analysis. Abbreviations: A: publications; B: country by income; C: country by region; D: number of people dying from HIV-related causes; E: number of new HIV infections; F: number of people living with HIV; G: prevalence of HIV among adults aged 15 to 49 (%); H: new HIV infections (per 1000 uninfected population); I: estimated antiretroviral therapy coverage among people living with HIV (%); J: estimated number of children living with HIV; K: estimated antiretroviral therapy coverage among children; L: estimated number of pregnant women living with HIV. * represents statistical significance (*p* value < 0.05).

Statistically significant positive correlations were identified between publication volume and several key indicators: the number of people dying from HIV-related causes (r^2^ = 0.88; *p* < 0.05; β = 0.5; SE = 0.1), new HIV infections per 1000 uninfected people (r^2^ = 0.97; *p* < 0.05; β = 0.7; SE = 0.15), and the estimated number of children living with HIV (r^2^ = 0.97; *p* < 0.05; β = 0.6; SE = 0.12). Furthermore, a statistically significant correlation was observed between country income levels and HIV prevalence among adults aged 15–49 years (r^2^ = 1.0; *p* < 0.05; β = 0.9; SE = 0.08), with prevalence significantly higher in low- and lower-middle-income countries (up to 16.6% in Uganda). The overall model showed an adjusted R^2^ of 0.96 and an F-statistic of 65.5 (*p* < 0.05), indicating a significant model fit. Notably, no correlation was identified between the volume of publications and HIV prevalence among adults, antiretroviral therapy coverage, or people living with HIV. Further correlations are presented in Figure 4.

## 4. Discussion

A bibliometric study effectively evaluates scientific activity, providing valuable information on scientific production, trends, and impact [30,31,32,33]. This study seeks to reveal gaps and hot topics to identify priority research areas [30,31,32,33]. The development of bibliometric studies has increased since 2020, and scientific databases have shown a substantial increase in this kind of study [30,31,32,33].

This study identified that most publications on stigma towards PLWH were original articles. The international co-authorship rate was 30%, and the average number of authors per article was five authors. Although the HIV epidemic began in the 1980s, it was in the 2000s that publications related to the stigma towards this condition began to increase. In 2022, there was more scientific production on this topic, which is a singular situation because it is now (40 years later) that this issue seems relevant to science [34,35].

The problem with stigma is that it is related to adverse effects on the health of people who suffer from it. Mainly because stigma leads to individuals being less likely to use prevention; hence, it is crucial to study the stigma towards PLWH, especially intending to find solutions to reduce this problem [36,37]. Although bibliometric studies on HIV/AIDS exist, they predominantly focus on antiretroviral treatment, pre-exposure prophylaxis, and biomedical interventions. These studies have contributed significantly to understanding treatment trends and clinical practices. However, research specifically examining attitudes and stigma related to HIV/AIDS through bibliometric approaches remains scarce.

While some systematic reviews have analysed stigma, they have typically been limited to specific geographic regions, such as the United States, and have primarily described the phenomenon without evaluating publication trends, research impact, or global collaboration patterns. In contrast, this study addresses a critical gap by providing a comprehensive bibliometric assessment focused on attitudes and stigma towards HIV/AIDS. It highlights trends, influential contributions, and areas requiring further exploration, offering a novel perspective distinct from previous research focused solely on biomedical aspects [37].

In 2015, Chambers et al. [36] in their study demonstrated that HIV-related stigma within the health context reflects society at large and is socially embedded and influenced by cultural values. Another study perceived that the stigma was associated with a higher prevalence of HIV transmission risk behaviours, no-use pre-exposure prophylaxis, and no HIV testing [37]. These associations underscore the need to explore research gaps and opportunities, aiming to close disparities and provide data to support evidence-based decision making.

Only three previous bibliometric studies had HIV as their main topic, but only one of them investigated the stigma towards HIV. That bibliometric study compiled 2509 documents in 2019, addressing the period from 1980 to 2017, which leaves out the great scientific production at a bibliometric level reported in the last five years and increases our analysis’s validity because our study actualises these data [38,39,40]. One of the main differences is that the study by Sweileh [40] analysed 2509 articles, while the present study analysed 15496 articles, and this difference is significant since both searches were carried out in Scopus. Another situation that can justify the significant differences in the results occurs because the search formulas differ in both cases [38,39,40,41]. Despite this, countries such as the United States of America, Canada, the United Kingdom, and South Africa continue to be the ones that lead the list with the highest publication rates [38,39,40,41].

The analysis of the h-index across journals, institutions, and countries provides critical insights into the influence and impact of HIV/AIDS stigma-related research. As shown in Table 2, the United States leads globally in research impact, with an h-index of 170, reflecting significant scientific contributions. Notably, institutions such as the University of California (h-index 95), Johns Hopkins University (h-index 70), and Columbia University (h-index 66) demonstrate strong academic leadership and output in this field. However, the data also highlight regional and economic disparities. While South Africa (h-index 86) and Canada (h-index 73) contribute meaningfully, low- and middle-income countries remain underrepresented despite bearing the highest HIV burden. This imbalance underscores the need for targeted research funding and collaborative networks to bridge knowledge gaps and contextualise interventions in resource-limited settings. Although the h-index is an indirect measure of impact, its utility lies in identifying influential work that can inform stigma reduction strategies. For example, widely cited research has already shaped policies promoting HIV testing, pre-exposure prophylaxis, and stigma reduction programs in key populations. Yet, translating this academic influence into practice requires focused efforts to implement evidence-based programs, particularly in under-researched regions.

Other differences between the bibliometric study of Sweileh [40] and the one presented here were that the main research topics in Sweileh’s study that topics were “Africa”, “women”, “adolescents”, “adherence”, “human rights”, “men who have sex with men”, and “South Africa”. In this study, the topics most used by researchers were “social psychology”, “social discrimination”, “sexual behaviour”, “homosexuality”, and “middle age”. In terms of authors, the five principal authors have changed since Sweileh’s publication [40]. However, this may be because that article considered the total citations, not the h and g-index, as in the present analysis. The leading journals in scientific production reported in both Sweileh’s manuscript and the present text were “Social Science & Medicine”, “AIDS and Behaviour”, “Journal of the International AIDS Society”, and “AIDS Education and Prevention” [40].

The correlational analysis revealed that countries with lower income levels had a reduced volume of publications on HIV/AIDS stigma, although this relationship was weak and not statistically significant. HIV prevalence also showed a slight negative correlation, indicating that higher prevalence is not directly associated with increased research on HIV stigma. The limited correlation between publication volume and income level may reflect a lack of funding in lower-income countries for investigating HIV stigma despite these countries often bearing a greater burden of the disease. This finding underscores the need for global approaches prioritising funding for public health research in low-income countries. Additionally, promoting international collaboration could help address these disparities.

According to the WHO, at the end of 2022, 39 million people were PLWH, two-thirds (25.6 million) from Africa [42]. The United Nations Programme on HIV/AIDS (UNAIDS) data showed that 29 million of the 39 million PLWH worldwide are receiving treatment. Access to antiretroviral therapy has significantly expanded in sub-Saharan Africa, Asia, and the Pacific, home to about 82% of all PLWH [43]. In 2022, the countries that reported the majority of the cases of HIV were South Africa, Mozambique, India, Uganda, and Brazil [43]. However, this study showed that the countries that carried out the most research on stigma and HIV are the United States, the United Kingdom, and South Africa. That is to say, among the countries with the highest number of new cases, only South Africa seems to consider stigma as an essential factor regarding HIV. As Greenwood et al. [44] and Cobos et al. [45] indicate, stigma is not a vague sociological notion; rather, it represents a real threat to public health, negatively impacting prevention, testing, and access to PLWH health services. Addressing stigma is essential to combat the HIV/AIDS pandemic effectively and should be a priority for the National Institutes of Health.

As a limitation of this study, one must note that utilising data deposited in the Scopus database as the unit of analysis links the quality directly to the records’ accuracy. Similarly, biases intrinsic to scientific publication exist, and these cannot be modified by the researchers executing this study.

Finally, we want to emphasise that stigma is a determining factor in the care of PLWH because it is a crucial contributor to poor HIV-related health outcomes. For example, some studies have noted that social stigma toward HIV is associated with a significant decrease in the likelihood of being tested for HIV, posing a significant barrier to screening among older adults, which is a significant obstacle for diagnosis and, therefore, for access to treatment, affecting the success of efforts to control HIV [46,47].

Now, if we consider the stigma on the part of professionals towards PLWH, this problem increases this crisis. Some authors have shown that the training rates on HIV care among professionals are low, and although the attitudes of professionals towards patients are generally positive, some professionals consider that PLWH could have acquired it through immoral behaviour; this type of negative attitude can be stigmatising and discriminatory and has been related to healthcare settings in which poverty, immigration, poor access to health, and deep-rooted religious beliefs converge [48,49]. Integrating strategies that enhance emotional intelligence and promote structured contact with people living with HIV/AIDS can complement educational programs, fostering empathy and dismantling entrenched stereotypes [50].

## 5. Conclusions

There has been a notable growth in research on attitudes and social stigma related to HIV/AIDS, with a significant transition in topics and research fields in the last four decades. Despite identifying a significant international collaboration network led by the United States and the United Kingdom, it could be more active, with greater inclusion of low- and middle-income countries, which would enhance the value and validity of the evidence. It was identified that there is no direct and equal correlation between the volume of evidence and global health HIV-related indicators, according to the most prolific countries and those with the highest burden of HIV disease. Published research has been predominantly qualitative, opening the possibility to explore novel quantitative or mixed-methods approaches for new research questions. These data serve as a foundation for the design of future studies related to attitudes and social stigma towards HIV/AIDS and for international collaborations involving diverse authors, institutions, and countries.

## Figures and Tables

**Table 1:** General characteristics of global research on HIV/AIDS attitudes and social stigma (N = 15.496).

	N	%
Timespan	1981–2024	-
Document Types		
Original articles	12,948	83.5
Reviews	1466	9.5
Editorials	293	1.9
Letters	789	5.1
Authors		
Authorships	40,403	-
Authors of single-authored docs (N = 40,403)	2438	6.03
Author Collaboration		
Single-authored documents	3656	-
Co-Authors per document	4.73	-
International co-authorships	-	30.66
Document contents		
Keywords	20,423	-
Journals	3023	-
Annual growth rate	-	8.98
Document average age (years)	11.1	-
Average citations per document	20.03	-

**Table 2:** Description, impact, and evolution of the most historically prolific affiliations and countries and the most cited articles on research about attitudes and social stigma towards HIV/AIDS.

Affiliations	Documents over Time				Documents	h-Index	Country
	1981–1990	1991–2000	2001–2010	2011–2024			
University of California	8	30	206	757	1001	95	USA
Johns Hopkins University	1	12	68	660	741	70	USA
University of Washington	0	1	52	553	606	48	USA
University of Toronto	2	6	61	504	573	54	Canada
Columbia University	3	13	84	361	461	66	USA
**Country**	**Documents over Time**				Documents *	**h-Index**	
	**1981–1990**	**1991–2000**	**2001–2010**	**2011–2024**			
USA	189	535	1349	5764	7837	170	
UK	24	84	307	1301	1716	94	
South Africa	1	8	282	1205	1496	86	
Canada	15	43	161	874	1093	73	
Australia	6	31	114	558	709	55	
**Article name (year of publication)**		**Journal characteristics**				**Participating countries**	**Citations**
		**Name**	SJR **	h index **	Quartile **		
HIV and AIDS-related stigma and discrimination: a conceptual framework and implications for action (2003) [20]		Social Science & Medicine	1.9	270	Q1	USA, UK	1705
Measuring stigma in people with HIV: Psychometric assessment of the HIV stigma scale (2001) [21]		Research in Nursing & Health	0.6	94	Q1	USA	1080
From Conceptualizing to Measuring HIV Stigma: A Review of HIV Stigma Mechanism Measures (2009) [22]		AIDS and Behavior	1.3	123	Q1	USA	716
Impact of HIV-related stigma on treatment adherence: systematic review and meta-synthesis (2013) [23]		Journal of the International AIDS Society	1.8	78	Q1	USA, Uganda	652
HIV-Related Stigma and Knowledge in the United States: Prevalence and Trends, 1991–1999 (2001) [24]		American Journal of Public Health	2.6	293	Q1	USA	645
Interventions to Reduce HIV/AIDS Stigma: What Have We Learned? (2003) [25]		AIDS Education and Prevention	0.5	79	Q2	USA	619
HIV testing attitudes, AIDS stigma, and voluntary HIV counselling and testing in a black township in Cape Town, South Africa (2003) [26]		Sexually Transmitted Infections	1.1	108	Q1	USA, South Africa	545
The Dimensionality of Stigma: A Comparison of Its Impact on the Self of Persons with HIV/AIDS and Cancer (2000) [27]		Journal of Health and Social Behavior	2.4	141	Q1	USA	544
An epidemic of stigma: Public reactions to AIDS (1988) [28]		American Psychologist	3.3	256	Q1	USA	489
A systematic review of interventions to reduce HIV-related stigma and discrimination from 2002 to 2013: how far have we come? (2013) [29]		Journal of the International AIDS Society	1.8	78	Q1	USA	472

SJR: Scimago Journal Ranking. * Individual production was counted; therefore, a document might be counted multiple times according to international collaboration. ** Metrics based on SJR 2023.

## Data Availability

The original contributions presented in the study are included in the article. Further inquiries can be directed to the corresponding authors.
